# An Insight Into the Factors Affecting the Prevalence and Natural History of Hepatitis D

**DOI:** 10.7759/cureus.43362

**Published:** 2023-08-12

**Authors:** Zaigham Abbas, Minaam Abbas

**Affiliations:** 1 Gastroenterology and Hepatology, Dr. Ziauddin University Hospital, Karachi, PAK; 2 Medicine, School of Clinical Medicine, University of Cambridge, Cambridge, GBR

**Keywords:** hepatitis d, cirrhosis, surrogate markers, natural history, transmission, prevalence, genotypes

## Abstract

Epidemiological studies and recent metanalyses addressing hepatitis D have reported a wide variation in the prevalence of the disease. Between 4.5% to 15% of all hepatitis B surface antigen (HBsAg) positive patients are thought to harbor the hepatitis D virus. The emergent variation in prevalence can be attributed to several factors. Unsurprisingly, published literature shows that the prevalence of the disease is higher in areas where aggregate viral hepatitis infections are endemic and amongst groups with high-risk practices facilitating the horizontal transfer. Meanwhile, the natural history of the disease is influenced by the genotype of the virus, the hepatitis D virus (HDV) RNA levels, HBV-HDV codominance, HBsAg titers, HBV genotype, nutritional status, HIV co-infection, and prior treatment. Together these factors contribute to the accelerated development of fibrosis and the increased risk of hepatocellular carcinoma. Superinfection with genotype 1 results in rapid progression to cirrhosis with lower rates of remission. Genotype 3 follows an aggressive course but shows a good response to interferon therapy. Other genotypes have better outcomes. The course of the disease leading to these outcomes can be tracked by HDV-specific models integrating clinical surrogate markers and epidemiological factors such as age, region, alanine aminotransferase (ALT), gamma-glutamyl transferase, albumin, platelets and cholinesterase, and liver stiffness.

## Introduction and background

Of all the immune-mediated viral hepatitis, hepatitis D is the most severe [1]. It often catalyzes rapid fibrosis, leads to the early development of hepatic decompensation, and accelerates oncogenesis [2]. There are at least eight hepatitis D virus (HDV) genotypes based on 14-38% sequence variation in genetic analyses [3]. These genotypes are associated with different long-term outcomes. Although the disease is aggressive in most patients, the course can vary, and many factors can influence disease progression and complications [4].

There are lacunae in the prevalence studies of hepatitis D and as a result, the meta-analyses integrating these studies also come to different conclusions [5-7]. The implementation of vaccination programs at different paces in various regions has changed the global epidemiology of the disease [8]. In addition, the threat of drug addiction and emigration from countries with high prevalence is leading to a resurgence of hepatitis D in countries where good control was previously achieved.

## Review

Patterns of prevalence

As of 2022, three large meta-analyses addressing the global burden of hepatitis D have been published [5-7]. The most recent study estimated that the worldwide prevalence of anti-hepatitis D virus (anti-HDV) antibodies was 4.5% in all HBsAg-positive subjects [5]. The number rose to 16.4% amongst those attending hepatology clinics for antiviral treatment and follow-up. At least 18% of HBsAg-positive patients with cirrhosis and 20% of those with hepatocellular carcinoma (HCC) harbored the virus [5]. The authors projected that 12 million people worldwide (0.16% of the general population in the studied epoch) were anti-HDV positive [5]. HBsAg-positive patients in Central Asia, Eastern Europe, and Western Africa were most likely to also be infected by HDV. Higher prevalence was seen in persons who inject drugs (PWID), patients undergoing hemodialysis, men who have sex with men (MSM), sex workers, and hepatitis C virus (HCV) or HIV-infected individuals [5].

A second meta-analysis argued for a higher prevalence of HDV in the general population, with 48-60 million infections worldwide (0.8% of the global population). It estimated a prevalence of 13% in HBsAg-positive patients. The study reported HDV-related fulminant hepatitis in over 26%, cirrhosis in over 25%, and HCC in nearly 20% of all HBV carriers. The odds ratio of detecting HDV in HBsAg-positive patients with chronic liver disease was 4.55 (when compared to asymptomatic carriers) [6].

Finally, a third systematic review investigating the global burden of hepatitis D virus infection in the period between 1977 and 2016 found an even higher prevalence of 0.98% in the general population, amounting to 62-72 million people (Table 1).

**Table 1:** Global HDV prevalence: current meta-analyses

	Stockdale et al., 2020 [5]	Miao et al., 2020 [6]	Chen et al., 2018 [7]
Number of studies/articles included	282	634	182
Period	January 1998-Jan 2019	Up to 2019	Jan 1977-Dec 2016
HDV prevalence worldwide %	0.16 (3.6-5.7)	0.8 (0.63-1.00)	0.98 (0.61-1.42)
HDV seroprevalence in HBsAg-positive individuals	4.5 (3.6-5.7)	13.02 (11.96-14.11)	14.57 (12.93-16.27)
Number of anti-HDV positive individuals globally (millions)	12	48-60	70
Estimate contribution of cirrhosis among HBsAg-positive people	18 (10-26)	25.77 (20.62-31.27) and HCC 19.80% (95% CI, 10.97-30.45)	NA
Estimate contribution of hepatocellular carcinoma among HBsAg-positive people	20 (8-33)	19.80 (10.97-30.45)	NA

HDV prevalence rose to 14.57% in HBsAg-positive patients [7]. As expected, a higher HDV prevalence was noted in the HBsAg-positive PWID population (37.57%) and in HBV patients undertaking high-risk sexual encounters (17%) [7].

The three studies show that areas of high seroprevalence of HDV encompass multiple continents [5-7]. Central and South Asia, Central and West Africa, Pacific Islands, the Amazon Basin, and Eastern Europe are key hotspots. The WHO described the Eastern Mediterranean Regional Office (EMRO) region, in particular, has a much higher rate of anti-HDV antibody positivity amongst their population [9]. Indeed, at least 16-18% of all Pakistani HBsAg-positive patients are infected by the HDV virus [10]. As with the global analysis, this is likely an underestimate as the prevalence of anti-HDV antibodies is up to 50% higher in HBV carriers attending hepatology clinics [9,11].

HDV genotypes

There are at least eight genotypes of HDV based on 14-38% sequence variation in genetic analyses. These genotypes are associated with different long-term outcomes (Table 2) [3,12-15].

**Table 2:** Distribution of HDV genotypes and impact on clinical outcome

Distribution	Clinical course
Genotype 1. Eastern Europe, Mediterranean and Middle East, Pakistan, Central and Northern Asia, North America, and North Africa	Rapid progression to cirrhosis with superinfection with lower rates of remission
Genotype 2. East Asia, the Yakutia region of Russia. Genotype 4. Taiwan and Japan	Fulminant hepatitis at the acute stage is less frequent. Fewer unfavorable outcomes (cirrhosis or HCC) at the chronic stage as compared to genotype 1
Genotype 3. The northern part of South America, especially the Amazon Basin	More progressive disease with poor prognosis. Better response to pegylated interferon compared to genotype 1
Genotypes 5-8. African countries and African migrant population	Milder disease compared to genotypes 1 and 3. Good response to interferon therapy

HDV genotype 1 is the most common and is globally distributed. The higher replicative efficiency may explain its widespread dissemination [16]. Meanwhile, HDV genotype 2 is mostly exclusive to regions in the Far East, while HDV genotype 3 is limited to the northern nations of South America. While a genotype distribution map is slowly starting to take shape, the situation continues to evolve and is complicated by emergent recombination events [17].

Discrepancies and conflict in the epidemiologic studies

The epidemiological situation in some neighboring countries of the same region and within the same country is very different. For example, Mongolia and Pakistan have a high burden of hepatitis D, but in neighboring countries, China and India, the prevalence of HDV is considered negligible [18]. This may be a consequence of underreporting given different national public health priorities. Using inappropriate disease cohorts can lead to a gross underestimation of the clinical implications of the infection. Meanwhile, studies from the Amazon basin population (Brazil and Peru) showed that the prevalence of HDV varied between 0, 30, 66, and 80% in highly endemic regions while the total seroprevalence in the respective countries was less than 1% [19]. In Pakistan, the prevalence in the districts of the central region, where three provinces meet, is higher than in the rest of the country [10]. This points to the need to investigate pockets of disease and the transmission factors underlying the endemicity. In addition, the results of studies conducted in high-prevalence hotspots cannot be extrapolated to the general population of the country (Table 3).

**Table 3:** Deficiencies and discrepancies in the epidemiological HDV data

Failure to recognize the issue and perform the screening for HDV
Lack of nationwide surveys
Evolving epidemiology, variable periods
Patchy information. The difference in denominators. Whether the study targets the general population, asymptomatic HBsAg carriers, high-risk groups or patients with cirrhosis visiting liver clinics
Prevalence may differ in immigrants versus vaccinated and different age groups
A small sample size of studies in the area with high HBsAg prevalence and ‘low HDV positivity’
Differences in the mode of spread in areas of low prevalence versus endemic areas
Genotypic variations affecting the natural history
The performance of different anti-HDV assays may vary
Most studies focus on anti-HDV and limited data on HDV RNA
Lack of funding and political will to conduct population-based studies due to low endemicity compared to HBV and HCV
Recent migration of refugees from high-prevalence areas to low-prevalence European islands

Transmission

HDV is primarily horizontally transmitted. The factors underlying the increased risk of infection are similar to those for HBV. However, the patterns of transmission in developed countries are changing due to the introduction of HBV vaccines, safer sex behaviors (precipitated by HIV fear, and the declining rates of risky sexual encounters due to public health campaigns), the widespread practice of proper sterilization of equipment, and the ease of availability of disposable Class II medical products such as syringes. Prevalence, and therefore transmission, nonetheless remains higher amongst PWID, hemodialysis recipients, MSM, sex workers, and immigrants from areas of high HDV endemicity [5,20]. The lack of universal screening of chronic hepatitis B patients for hepatitis D fails to detect infection in these countries and may facilitate transmission in high-risk populations.

Risk factors in endemic areas are mainly nosocomial. Unsafe needle and syringe handling, the reuse of unsterilized equipment and needles in a medical setting and beyond (barber and tattoo shops), and quackery are the main causes of the spread of infection [21]. These modes of transmission are coupled with a lack of treatment options and reduced access to healthcare services due to poverty and have led to the rapid proliferation of disease.

Perinatal HDV transmission remains debatable. A Saudi study following 185 HBsAg-positive pregnant women (9.7% anti-HDV positivity) found that of the 17 infants born to anti-HDV-positive mothers, none had signs of HDV infection at seven months [22]. In a French cohort, 22/742 of the HBV-infected women studied were infected with HDV. Of the 36 children from these patients, two-thirds were protected after passive-active immunization, 10 were neither infected nor protected, and two were either chronically or acutely HBV infected. Anti-HDV antibodies were negative in all [23]. Therefore, it has been suggested that HBV/HDV simultaneous transmission from mother to child is unlikely or at least exceedingly rare. Intra-familial transmission does occur but this is likely through close personal contact and unsafe practices over an extended period [24].

**Figure 1:**
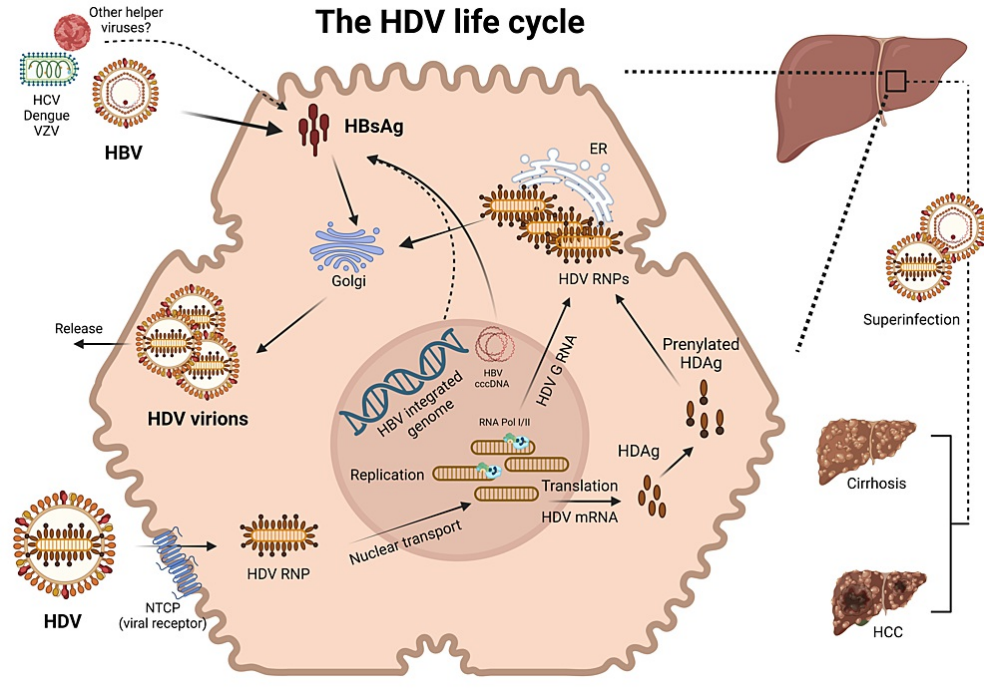
Hepatitis D virus (HDV) life cycle HDV viral entry is facilitated by the Sodium taurocholate co-transporting polypeptide (NTCP) binding of the HBsAg. Replication of the HDV genome (HDV G) is nuclear and is mediated by RNA Pol I/II. HDV mRNA is used to make hepatitis D antigen (HDAg). The HDAg is prenylated and along with HDV G, is used to assemble the HDV ribonucleoprotein (RNP) in the endoplasmic reticulum (ER). The HDV RNP is enveloped in the Golgi by HBsAg. Expression of cccDNA and HBV integrated into the human genome may provide HBsAg. Other helper viruses may also provide envelope proteins. Superinfection increases the risk of cirrhosis and hepatocellular carcinoma compared to HBV monoinfection.

The central dogma in the field is that HBV is necessary for the transmission of the virus. In the absence of circulating HBsAg, expression of HBsAg may occur from the HBV covalently closed circular DNA (cccDNA) or from integrated HBV in the host genome [25,26]. It has now been shown that hepatitis C, vesicular and dengue viruses can also help package the HDV ribonucleoprotein (RNP) to make infectious particles. However, these studies are limited to in-vitro and in-vivo observations and the clinical implications of these finds are unclear (Figure 1) [27].

Coinfection versus superinfection

Co-infection refers to simultaneous infection with HDV and HBV leading to the occurrence of acute hepatitis D and B. Superinfection is the HDV infection of a chronically infected HBV carrier [28].

Co-infection is usually acute, self-limiting, and clinically indistinguishable from acute HBV infection. These patients are distinguished by the high titer of anti-hepatitis B core IgM (anti-HBc IgM) antibodies. As with HBV mono-infection, less than 5% of patients progress to chronic HBV and/or HDV infection. However, co-infection can cause severe hepatitis with a high probability of developing fulminant hepatitis [6]. The risk of this occurrence is higher in the PWID population.

In superinfection, anti-HBc IgM antibodies are usually absent. Only a minority of patients clear HDV spontaneously. Acute hepatitis upon infection is severe with a short incubation period and progresses to chronic hepatitis D in over 77% of cases. According to a meta-analysis with a random-effects model, HDV replication dominance occurs in a majority of patients (69.28%), while codominance (27.56%) and HBV replication dominance (3.16%) are less common [6]. Superinfection is more likely to cause acute-on-chronic liver failure or severe chronic active hepatitis rapidly progressing to cirrhosis [29]. Indeed, HDV replication in superinfected individuals leads to cirrhosis and HCC at annual rates of 4% and 2.8%. The persistence of HDV is the only predictor of liver-related mortality in this population of patients [30].

Development of cirrhosis

Chronic infection causes an accelerated progression to fibrosis and early decompensation in cirrhotic patients [31]. In 70% of all co-infected patients, cirrhosis occurred within 5-10 years of HDV infection and indeed can occur as early as two years after initial infection. This corresponds to a three-fold increase in disease progression when compared to HBV mono-infected patients [32]. Cirrhosis develops at an annual rate of 4% [33], while decompensation in HDV cirrhotics occurs at an annualized rate of 2.6-3.6% [34]. The severity of disease progression is dependent on the host and viral factors and is discussed below [35]. The risk of liver-related events increases 3-4 folds in patients with viremia than in those without circulating HDV RNA [36]. However, indolent courses and asymptomatic patients have also been reported [37,38]. A quarter of chronic hepatitis D patients achieve a spontaneous ≥2 log_10_ decrease in HDV RNA and in 20% HDV-RNA becomes undetectable during the natural course of the disease [39].

Extrahepatic complications

Interestingly, HDV-infected patients rarely have any extrahepatic complications. This is in sharp contrast to patients with hepatitis B or C [40].

HDV and hepatocellular carcinoma (HCC)

The estimated annual incidence of HCC in HDV patients with cirrhosis is 2.6-2.8% [41]. Hepatic decompensation is by far a more common clinical endpoint rather than the development of HCC, but when compared to mono-infected HBV patients, the risk of HCC is higher [34,42], with a pooled OR of 1.28. With heterogeneity corrected for in the meta-analysis underpinning this data, the pooled OR rises to 2.77 [43]. Oxidative stress because of severe necro-inflammation likely triggers HCC oncogenesis. Epigenetic processes such as DNA methylation and histone modifications are likely to also contribute to the proliferation and evolution of the tumor. Moreover, several studies have shown that genomic instability may have a part to play as genes associated with DNA damage, repair, and replication are often upregulated in these patients [34,42,44].

HDV is thought to induce HCC through mechanisms distinct from HBV. Inflammation and cirrhosis have a central role. Indeed, at least one study has shown that HCC patients with HDV have a decreased liver size compared to those with HBV monoinfection, where the liver size was either normal or increased. Moreover, the HDV patients in this study had lower platelets and larger varices, pointing to a more severe cirrhotic state. This corresponded with a more severe presentation of HCC, with a TNM Stage III/IV, multifocal tumors, and alpha-fetoprotein (AFP) > 1000 IU/ml in these patients [45].

Possible factors influencing disease severity

Several factors can influence the severity and course of the disease (Table 4).

**Table 4:** Possible factors affecting the severity of hepatitis D disease

HDV related	HBV related	Host related
HDV RNA levels	HBV-HDV co-dominance	Age
HDV genotype	HBsAg titer?	HDV ‘carrier state’ (immune response)
	HBV genotype?	Nutritional status
		HIV co-infection
		Previous HDV treatment

HDV RNA Levels

The levels of HDV RNA correspond to the probability of developing cirrhosis and HCC [46,47]. Once cirrhosis has developed, the amount of HDV RNA has little predictive value of further progression [46].

HBsAg Levels

There is a complex interaction between the hepatitis B and D viruses during the disease. Suppression of hepatitis B virus replication by the hepatitis D virus is associated with increases in HBsAg levels, and these levels correlate with HDV viremia [48]. HBsAg serum levels in hepatitis D are a positive predictor of response to interferon-alpha therapy [49], which significantly suppresses both HDV RNA and HBsAg.

Interestingly, there is emerging evidence that HDV might be able to persist in hepatocytes even in the absence of the helper virus. It is hypothesized that the partial or complete integration of HBV into the human genome can serve as a source of HBsAg and can sustain the replication of HDV [25,26].

HDV Genotypes

HDV genotypes may influence the disease severity (Table 2).

HBV and HDV Genotype Interactions

The genotypic distribution of the hepatitis B and D viruses is highly varied. There are eight HDV genotypes and 10 HBV genotypes and several subtypes. Large studies are needed to assess the effect of various permutations of HBV/HDV genotypes on disease severity [50]. As a starting point for this investigation, it is known that monoinfection with HBV genotypes C, D, and F compared to A and B is more likely to cause cirrhosis or HCC [51]. These genotypes could synergize with certain genotypes of the HDV. Indeed, HBV genotype F in combination with HDV genotype 3, has been reported to accelerate fulminant hepatitis [52].

Dominance vs Codominance

Longitudinal assessment of viremia reveals a complex interaction between HBV and HDV. HDV suppresses the replication of HBV in most individuals superinfected with HDV [53]. Patients with HDV genotype 1 (HDV-1) dominant infections are likely to have slower disease progression than those with co-dominant infection [54].

The Hepatitis Delta International Network (HDIN) has shown that while most patients with chronic HDV are HBeAg-negative (77%) [55], South Asian patients are more likely to be HBeAg positive (35.2% vs 10-16% globally). A total of 60.8% of this population is also HBV DNA positive. In turn, this observation correlates strongly with the fact that patients from this region are younger (32.7 years) than the rest of the world. It may be the case that codominant infections facilitate a speedier progression in this population.

Age at the Outset of Infection

The age of infection varies from region to region and the mean age of infection has been reported as 36.7 years [55]. Horizontal transmission results in childhood or early teenage infection in sub-Saharan Africa and Pakistan, whilst those in the Mediterranean are found to be infected later in their adult life [53]. An analysis of data from 1576 patients from the HDIN registry showed that the patients from South Asia and Eastern Europe were younger than the patients from Central or Southern Europe [55]. The question arises whether age at infection can impact clinical outcomes [56].

It is unclear whether children seem to control HDV well. An Italian study showed that HDV-infected children typically had minimal symptoms with a correspondingly high rate of HBV replication in the liver [57]. On the other hand, a small study investigating Pakistani pediatric patients (aged up to 18 years) found that HBV DNA and HBeAg were detectable in over 50% of HDV patients. Despite this, alanine aminotransferase (ALT) was elevated in almost all patients and there was no difference in the severity of disease in HBeAg-reactive and HBeAg-nonreactive HDV patients. Indeed, of the six patients with decompensation, five were HBV DNA positive [58]. Moreover, HDV-infected children in this study progressed to significantly worse outcomes than those with HBV mono-infection alone (matched controls). The study showed that children are unlikely to fare well with HDV infection. Co-dominance of HBV may also have a role in this population and combined with the young age of infection, could lead to early decompensation.

Young adults (18-25) have also been shown to be at severe risk of disease progression. Approximately a third of patients had cirrhosis at presentation, suggesting an aggressive course of disease [59]. HBV DNA was detectable in 70% of the cases, indicating a preponderance of codominant infections. It remains unclear whether age predisposes to codominance or if age independently confers a disadvantage to clinical outcome.

Nutritional Status

Data analyzed from the HDIN registry showed that compared to the rest of the world, more patients from South Asia (40% vs less than 20%) had low albumin (<3.5 g/L) [55]. This is correlated with worse outcomes in this subgroup.

Hepatitis D and HIV Infection

Disease progression is slower in HIV-HDV patients as compared to HIV-HCV and HIV-HBV coinfected patients [60]. This is evidenced by the longer time free from liver decompensation or death.

Influence of Previous Treatment

Patients treated with interferon seem to benefit from the therapy. Although the sustained loss of HDV RNA is not usually achieved, normalization of liver enzymes often occurs and the quality of life improved [61]. Pegylated interferon seems to similarly have a positive impact on the treatment of hepatitis D [62]. The HIDIT-1 study has shown that after pegylated interferon therapy, event-free survival is better in patients with an HDV-RNA response at 24 weeks post-treatment than in patients who had positive HDV-RNA at 24 weeks [63]. Therefore, a reduction in the viral load translates into better outcomes.

Clinical markers of disease progression

Various surrogate clinical markers can be used to track the course of a chronic HDV infection and predict cirrhosis.

Delta Fibrosis Score (DFS)

The scoring utilizes cholinesterase, albumin, gamma-glutamyl transferase (GGT) levels, and the age of the patient [64]. Low cholinesterase, low albumin, high GGT, and older age have predictive value for fibrosis.

The Delta-4 Fibrosis Score (D4FS)

D4FS utilizes GGT, platelet count, ALT, and liver stiffness measurement (LSM) and can accurately detect cirrhosis in chronically infected patients [65].

Baseline Event-Anticipation (BEA) Score

BEA score can also be used to predict the risk of developing liver-related morbidity and mortality in patients infected with HDV [66]. Factors associated with poor long-term outcomes include male gender, age >40, Southeast Mediterranean origin, platelet counts ≤100 x 10^3^/ml, platelet counts ≤50 x 10^3^/ml, high bilirubin, and high INR values ≥ 1.2. Each factor scores a point and a high BEA score corresponds to worse outcomes.

Serum Cholinesterase

Serum cholinesterase levels alone can also predict liver reserves in hepatitis D and correlate with the Child-Turcotte-Pugh (CTP) score, Model of End-stage Liver Disease (MELD) score, and BEA scores [67].

Model for the Severity of Hepatitis D in Young Adults

A three-variable model based on spleen size, albumin level, and platelet count and a two-variable model based on spleen size and platelet count can be used to predict clinical outcomes in young adults with HDV infection [59].

Screening and prevention

According to the AASLD guidelines, testing for HDV is recommended in HBsAg-positive patients who have risk factors for HDV infection or unexplained elevated transaminases [68]. The APASL guidelines recommend HDV testing in HBV patients with chronic liver disease [69]. The EASL guidelines support universal testing for HBsAg-positive patients [70]. Accumulating data supports the EASL’s position and reflex testing for HBsAg-positive individuals is recommended. Patients should be tested for anti-HDV antibodies, followed by confirmatory testing for HDV-RNA [71]. This can improve detection rates for HDV-infected individuals and indeed a recent study has shown that testing all HBsAg-positive patients for HDV can result in a five-fold increase in HDV diagnoses [72].

Preventing the transmission of HBV will prevent the global proliferation of the delta virus. If a person is immunized against HBV and does not get HBV, they cannot contract the delta virus. The need of the hour is to implement WHO’s recommendation to immunize all infants within 24 hours of birth (followed by a further three doses as part of the expanded immunization program). Pregnant women with high HBV DNA levels (200,000 IU/mL) and/or HBeAg positivity should receive tenofovir prophylaxis to prevent transmission to their newborns [73].

## Conclusions

Epidemiological studies have identified large differences in the prevalence of hepatitis D. Between 4.5% and 15% of all hepatitis B surface antigen (HBsAg) positive patients carry the hepatitis D virus. The natural history of the disease is influenced by viral genotype, HDV RNA levels, HBV-HDV codominance, HBsAg titers, HBV genotype, nutritional status, HIV co-infection, and prior treatment. The disease progression can be followed by HDV-specific models that integrate clinical surrogate markers and epidemiological factors such as age, region, alanine aminotransferase, gamma-glutamyltransferase, albumin, platelets and cholinesterase, and liver. Universal screening of HBsAg-positive individuals for HDV will improve detection rates. Preventing the transmission of HBV will prevent the worldwide spread of the Delta virus.
